# The economic burden of diabetes-related visual impairment and blindness in Saudi Arabia

**DOI:** 10.1186/s13561-026-00721-3

**Published:** 2026-01-24

**Authors:** Gihan Hamdy Elsisi, Abdullah Alhumaidan, Abdulrahman Alturaiki, Hana Abdul Kareem, Nada Abu-Shraie, Ramzi Al Judaibi, Saleh Alshehri

**Affiliations:** 1 Economics, American University in Cairo, Cairo, Egypt; 2 King Khaled Eye Specialist Hospital (KKESH), Riyadh, KSA, Saudi Arabia; 3 Ministry of National Guard - Health Affairs, Riyadh, Saudi Arabia; 4 Jeddah Eye Hospital, Jeddah, Saudi Arabia

## Abstract

**Objectives:**

Our main objective is to measure the economic impact of managing diabetes-related visual impairment and blindness in the Kingdom of Saudi Arabia (KSA) to inform policy and resource allocation for effective disease management and prevention.

**Methods:**

We develop a prevalence-based cost of illness model using Microsoft Excel to assess the economic impact of treating diabetic-related visual impairment and blindness over a one-year period from societal perspective. The model categorizes costs according to the severity level of the condition: mild, moderate, and severe visual impairment and blindness. It encompasses direct medical expenses such as those for drug acquisitions, surgical procedures, healthcare resources, monitoring, visual aids, and psychological care, as well as indirect costs such as productivity losses in the financial year 2024. The model’s outputs provide a cumulative and detailed breakdown of the costs associated with visual impairment and blindness in KSA. Sensitivity analysis was conducted.

**Results:**

Our economic model shows that the total economic burden of diabetes-related visual impairment and blindness in KSA in one year is SAR 150,324,912,186 ($ 81.2 billion). This cost is divided into direct and indirect medical costs estimated at SAR 101 billion ($ 54.6 billion) and SAR 49 billion ($26.5 billion), respectively. The major component is medication costs, along with patients’ productivity loss.

**Conclusion:**

The significant economic repercussions of diabetes-related visual impairment and blindness across individuals, households, and the Saudi healthcare system impose a substantial financial burden. These findings highlight the urgent need for investment in innovative interventions and screening programs aimed at mitigating these costs, enhancing accessibility to essential treatments, improving the quality of life of affected individuals and families, and achieving sustainable healthcare and socioeconomic development goals in the Kingdom.

**Supplementary Information:**

The online version contains supplementary material available at 10.1186/s13561-026-00721-3.

## Background

Visual impairment, resulting from disease or insufficient refractive correction, limits the eye’s functionality and can hinder daily activities. This impairment is a significant global health issue that potentially diminishes overall quality of life by affecting personal well-being, psychological health, mobility, and social interactions, leading to socioeconomic losses. [1, 2] Thus, visual impairment and age-related eye diseases negatively impact economic and educational opportunities, lower quality of life, and increase the risk of mortality [3]. Primary age-related eye diseases include cataracts, age-related macular degeneration, glaucoma, and diabetic retinopathy [4]. 

A comprehensive meta-analysis that included studies from 98 countries identified uncorrected refractive error as the primary cause of severe visual impairment, affecting 116.3 million people. The other causes identified were cataracts (52.6 million), age-related macular degeneration (8.4 million), glaucoma (4.0 million), and diabetic retinopathy (2.6 million). Meanwhile, cataracts were found to be the leading cause of blindness, affecting 12.6 million people, followed by uncorrected refractive error (7.4 million) and glaucoma (2.9 million) [5]. 

A retrospective cross-sectional study conducted in the Kingdom of Saudi Arabia (KSA) in 2018, examined 195 consecutive older adults at the outpatient ophthalmology clinic of King Abdul-Aziz University Hospital. The study found a high prevalence of visual impairment (14.9%) among Saudi adults aged 40 years and over. The primary factors linked to visual impairment were glaucoma and diabetic retinopathy, both of which can cause irreversible vision loss [6]. 

Visual impairment imposes various costs beyond the usual healthcare expenses. Affected individuals may experience reduced job opportunities, increased absenteeism, and lower productivity when adequate support is lacking. Caregivers and family members also experience productivity losses while fulfilling their crucial roles. Moreover, when individuals’ productivity declines, the societal benefits and values associated with their contributions are reduced [7]. 

Diabetes mellitus (DM) affects millions of people worldwide, and projections show a continuous rise. In KSA, the prevalence of DM is notably high, reaching 17.6% in 2015, the highest in the Middle East and North Africa (MENA) region. Diabetic retinopathy (DR) is a serious complication of diabetes that can lead to vision loss among adults worldwide, affecting approximately 34.6% of individuals with diabetes [8] and accounting for 2.5% of global blindness [9]. 

Studies conducted between 2015 and 2021 have indicated DR prevalence rates ranging from 6.25% to 54.6% among populations with diabetes in various regions of KSA. These findings underscore the escalating impact of diabetes on eye health in the country, reflecting the broader trends of increasing diabetes prevalence nationwide [10] and highlighting the need for screening, prevention, and disease management guidelines and protocols.

Despite improvements in healthcare services and access to free health coverage in KSA, barriers to timely DR screening and treatment contribute to delayed diagnosis and poor prognosis. A cross-sectional study conducted at King Khaled Eye Specialist Hospital in KSA involving 338 patients who had not previously received ocular intervention revealed that most patients were over 50 years old (60.4%) and that reduced vision was the primary presenting symptom (81%). The study identified the lack of patient knowledge about the importance of DR screening and issues within the healthcare system as major contributors to the delays in diagnosis and management. Despite improvements in DR screening and treatment in KSA, socioeconomic and healthcare system challenges continue to hinder optimal management and outcomes [11]. 

Hence, eye health can be viewed as a multifaceted development issue. Taking steps to improve population eye health and address visual impairment has the potential to significantly support the achievement of the sustainable development goals [12, 13].

In high income countries, a systematic review of 22 studies conducted to assess the economic burden of visual impairment and blindness reported that hospitalization and use of medical services were the largest contributor to direct medical costs. The mean annual expenses per patient were found to be US$ purchasing power parities (PPP) 12,175–14,029 for moderate visual impairment, US$ PPP 13,154–16,321 for severe visual impairment and US$ PPP 14,882–24,180 for blindness, almost twofold the costs for non-blind patients [14].

Decision makers need a comprehensive assessment of economic burden for those patients to improve population eye health and support the achievement of the sustainable development goals. We perform this economic study to understand the financial impact of diabetes-related visual impairment and consequently provide a reference for resource allocation and policymaking.

## Methods

### Study design

We constructed our model based on the societal perspective in KSA. To support our economic study, we conducted a systematic literature review from year 2000 to 2024 to gather all pertinent cost and resource utilization data related to the treatment of visual impairment and blindness associated with diabetes.

Furthermore, we addressed gaps in knowledge concerning local clinical guidelines and practices for major diseases causing visual impairment by meeting practicing Saudi ophthalmologists. A well-structured questionnaire was used by an expert panel to validate the assumptions and inputs incorporated into our model.

### Model design

**Fig. 1:**
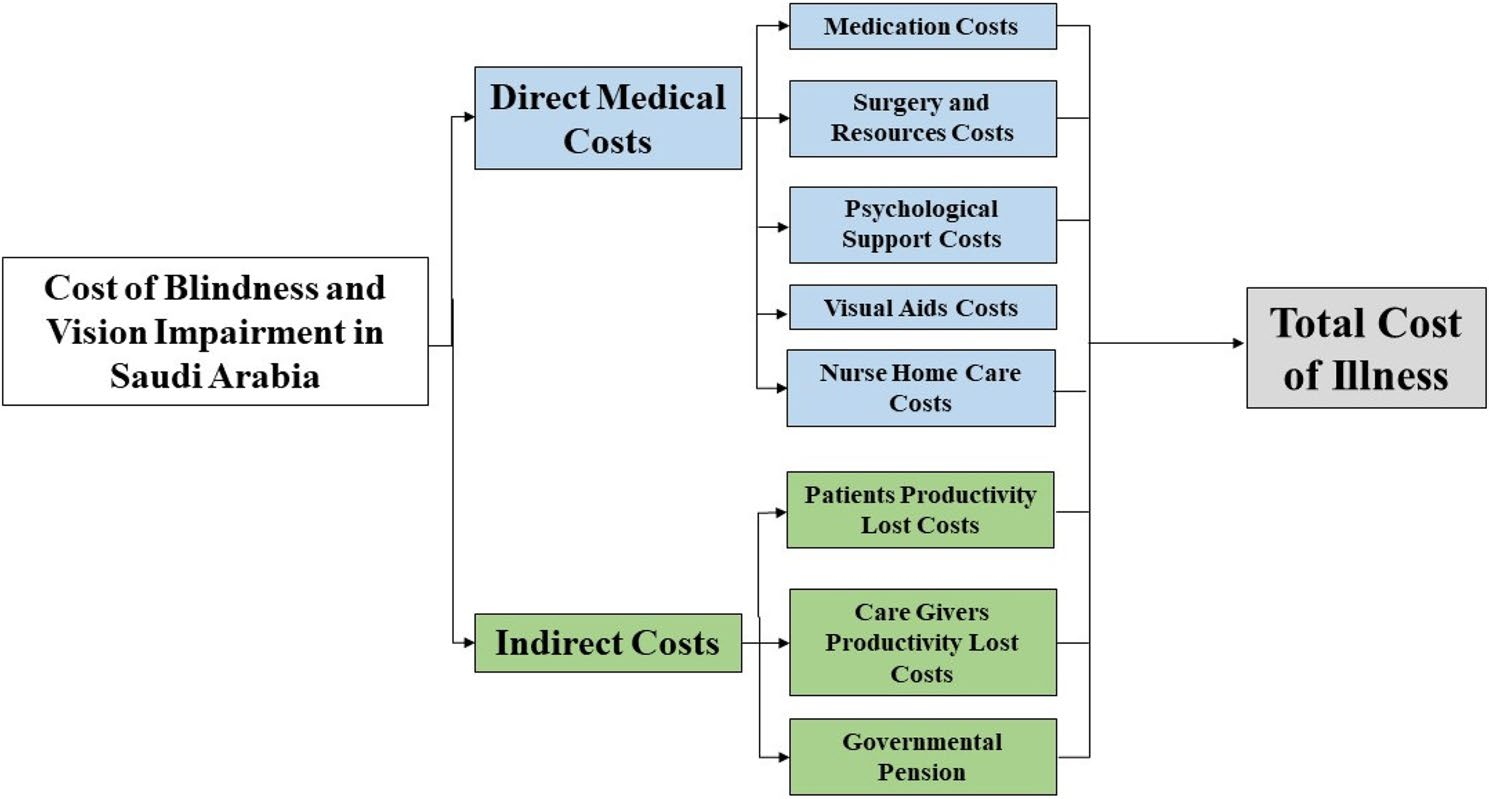
Cost of Illness Model

We built our prevalent cost of illness model in Microsoft Excel to evaluate the costs associated with the management and treatment of diabetes-related visual impairment and blindness in adult KSA patients over a one-year time horizon. The cost of illness model structure is illustrated in Fig. 1.

The model clearly separated the management and associated costs of mild, moderate, and severe diabetes related to visual impairment and blindness. Moreover, the model included direct medical costs (drug acquisition, surgeries, healthcare resources, monitoring, psychological care, visual aids, and homecare nursing costs) and indirect costs (productivity costs and government pension). The model’s outputs include the cumulative and breakdown of the costs associated with diabetes-related visual impairment and blindness in KSA.

There are some assumptions that need to be mentioned. The model adopted a one-year time horizon and a societal perspective, assuming that all direct medical and indirect productivity costs occurring within this period were captured. Treatment pathways for diabetic retinopathy and maculopathy were assumed to follow standardized clinical protocols validated by a Saudi expert panel, including fixed annual frequencies of anti-VEGF injections, corticosteroid injections, laser therapy, vitrectomy, follow-up investigations, and monitoring tests. Adverse event risks (endophthalmitis, retinal detachment, cataract formation) were applied uniformly to all treated patients based on clinical trial data. The model assumed that R1, R2, M1, and R3 patients require visual aids, whereas R4, M2, and blind individuals require psychological support and annual home-care nursing. Productivity loss estimates were based on expert-validated assumptions that 80% of mild, moderate, and severe visually impaired individuals are employed, and that their annual absenteeism equals 14, 21, and 28 days, respectively. Caregivers of moderate and severe cases were assumed to lose 10 and 28 working days, respectively, while sight-threatening disease was assumed to cause extensive work loss (261 days).

### Population

The model’s target population was determined from the published literature and according to the feedback of our expert panel comprising three Saudi ophthalmologists from Retina Department, King Khaled Eye Specialist Hospital, Riyadh, and Jeddah Eye Hospital, Jeddah, KSA. Insights were obtained using three rounds of meetings by employing a quasi-Delphi panel approach. The target population within our model comprised individuals diagnosed with diabetes-related blindness and visual impairment in KSA and treated, as shown in Table 1. The model’s target population was based on the total number of KSA adults aged ≥ 35 years who had been diagnosed with diabetes since May 2017 [15–18]. 

**Table 1:** Target Population within the Cost of Illness Model

Inputs	Probability (%)	Population	Category	Reference
Cost of Illness Model Population				
Adult Saudi Arabia Population (≥ 35 years) of the total population	33%	12,830,572	-	[14, 15]
Diabetic Patients in KSA	17.7%	2,271,011.24	-	[16]
Blindness among Diabetic Individuals	3.3%	74,943.37	-	[17]
The prevalence of any retinopathy in Diabetic Population	34.50%	783,498.88		[17]
The prevalence of any maculopathy in Diabetic Population	20.30%	461,015.28		[17]
The prevalence of any retinopathy and/or maculopathy in Diabetic Population	36.8%	835,732.14	-	[17]
Prevalence of Mild Non-Proliferative DR (R1)	22.1%	337,037.96	Mild	[17]
Prevalence of Moderate Non-Proliferative DR (R2)	5%	76,252.93	Moderate	[17]
Prevalence of Severe Non-Proliferative DR (R3)	3.9%	59,477.29	Severe	[17]
Prevalence of Proliferative DR (R4)	3.5%	53,377.05 27,391.11	Severe/Sight Threatening	[17]
Diabetic Maculopathy (M1)	4.4%	67,102.58	Moderate	[17]
Diabetic Maculopathy (M2)	15.9%	242,484.32	Severe/Sight Threatening	[17]

*KSA* Kingdom of Saudi Arabia, *DR* diabetic retinopathy

The prevalence of blindness and visual impairment due to diabetes in KSA was derived from a study conducted in the region that involved 3,052 individuals and used the Scottish DR grading system [19]. The major visual diseases due to diabetes that causes visual impairment in KSA were determined by our expert panel, and their prevalence rates were based on published literature [19]. 

Disease severity was classified as follows for model development: individuals with mildly impaired vision in our model were patients with mild nonproliferative DR (R1) while individuals with moderately impaired vision were patients with moderate nonproliferative DR (R2) and stage 1 diabetic maculopathy (M1). Nonproliferative DR (R3) was considered severe. Proliferative DR (R4) and stage 2 diabetic maculopathy (M2) were regarded as sight-threatening diseases. A corrected factor was applied to account for the overlap in diabetic population in patients having combined Maculopathy and/or Retinopathy:

Correction Factor = (Any Retinopathy AND/OR Maculopathy)/(Any Retinopathy + Any Maculopathy).

This factor is then applied after that to each prevalence. This factor was applied to avoid double-counting patients who have both DR and maculopathy.

### Clinical inputs

In the model, the major diseases causing diabetes-related visual impairment in KSA were diabetic retinopathy and diabetic maculopathy. These diseases were managed according to published treatment protocols and expert panel feedback. The treatments, management protocols, and resource utilization are described in Annex (1).

The probabilities of adverse events associated with disease management were extracted from published clinical trials [20–22] and are presented in Table 2.

**Table 2:** Adverse Events Probabilities

Parameter	Probability	Reference
Adverse Events		
Anti-VEGF AEs for Diabetic Retinopathy and Macular Edema		
Endophthalmitis probability after Anti-VEGF	0.035%	[18]
Retinal Detachment after Anti-VEGF	0.013%	[19]
Diabetic Retinopathy Management AEs		
Cataract Surgery after Vitrectomy for Retinopathy Management	48.04%	[20]

*AEs* Adverse Events, *VEGF* vascular endothelial growth factor

### Costs

All the unit costs utilized in the model are listed in Table 3. The model included direct medical costs (drug acquisition, surgeries, healthcare resources, monitoring, visual aids, and psychological care costs) and indirect costs (productivity costs and government pension). Medication unit costs were obtained from the Saudi Food and Drug Authority’s drug list database while healthcare resource unit costs were retrieved from Saudi hospitals’ price lists and Ministry of Health records. These costs were measured in SAR in 2024 and converted to International dollars by using the purchasing power parity exchange rate for results comparability.

**Table 3:** Unit Costs in the Model

Item	Unit Costs	
Medications Unit Costs		I$
Intravitreal injections - LUCENTIS 10MG-ML INTRAVITREAL Injection (Anti-VEGF)	SAR 2,942.40	$1,590.49
MINIMS ATROPINE SULPHATE - Dilating Eye Drops	SAR 1.26	$0.68
AB Eye Drops - VIGAMOX	SAR 26.60	$14.38
Topical Corticosteroids - NEOPRED – P	SAR 6.40	$3.46
Vancomysin – 500 mg Injection	SAR 45.15	$24.41
Vancomysin − 1000 mg Injection	SAR 76.90	$41.57
Ceftazidime 1000 mg (Intravitreal injection)	SAR 51.55	$27.86
Ceftazidime − 2 g	SAR 95.40	$51.57
TRIAMCINOLONE ACETONIDE 4 mg	SAR 295.05	$159.49
Average Resources Unit Costs		
Intravitreal Injections	SAR 2,000.00	$1,081.08
Laser Photocoagulation	SAR 1,200.00	$648.65
Optical coherence tomography (OCT)	SAR 380.00	$205.41
Ophthalmologist Visit - HCP Visit	SAR 345.00	$186.49
Fundoscopic examination	SAR 60.00	$32.43
Visual Acuity	SAR 180.00	$97.30
Fluorescein angiography	SAR 480.00	$259.46
Visual Field Test	SAR 180.00	$97.30
Pars Plana Vitrectomy (PPV)	SAR 3,200.00	$1,729.73
Aqueous and Vitreous Sampling	SAR 1,600.00	$864.86
Hospitalization Cost	SAR 600.00	$324.32
Vitrectomy	SAR 3,400.00	$1,837.84
Intra-occular Pressure Measurement	SAR 80.00	$43.24
Gonioscopy	SAR 40.00	$21.62
Optical devices such as spectacles, magnifiers, telescopes, and electronic magnification aids	SAR 3,000.00	$1,621.62
Phacoemulsification Surgery	SAR 4,400.00	$2,378.38
Intra-occular Lens	SAR 2,800.00	$1,513.51
Refraction Test for selection of IOL	SAR 520.00	$281.08
Biometry (A-Scan)	SAR 520.00	$281.08
Ocular Surface Evaluation	SAR 104.00	$56.22
Pupil Size and Dynamics	SAR 104.00	$56.22
Corneal Topography	SAR 160.00	$86.49
IOL Position and Stability Evaluation	SAR 1,500.00	$810.81
Ocular Surface Assessment	SAR 104.00	$56.22
Psychologist Support - HCP Visit	SAR 345.00	$186.49
Nurse Home Care Salary per Year	SAR 37,000.00	$20,000.00
Productivity Costs		
KSA Salary for Severe Vision Impaired Individuals and Blind Individuals per Month	SAR 4,000.00	$2,162.16
Working Individuals with Mild/moderate/Severe/Sight threatening Vision Impairment.	80%	Non applicable
Total Productivity Lost (Absenteeism) Annual Days for Mild Vision Impaired Individual	14 Days	
Total Productivity Lost (Absenteeism) Annual Days for Moderate Vision Impaired Individual	21 Days	
Total Productivity Lost (Absenteeism) Annual Days for Severe Vision Impaired Individual	28 Days	
Total Productivity Lost (Absenteeism) Annual Days for Moderate Vision Impaired Care-Giver	10 Days	
Total Productivity Lost (Absenteeism) Annual Days for Severe Vision Impaired Care-Giver Individual	28 Days	
Total Productivity loss for Sight Threatening DR	261 Days	
Average working days in Saudi Arabia - Week	5 Days	
Average working days in Saudi Arabia - Month	22 Days	

*KSA* Kingdom of Saudi Arabia, *DR* diabetic retinopathy, *IOL* Intraocular Lens

In reference to the local Delphi panel, only individuals with mild (R1), moderate (R2, M1), and severe (R3) diabetes-related visual impairment needed visual aids while individuals with blindness and sight-threatening severe (R4 and M2) diabetes-related visual impairment needed psychological support and homecare nurses.

In our model and based on our Delphi panel, we assumed that only 80% of individuals with mild (R1), moderate (R2, M1), and severe (R3) diabetes-related visual impairment were working and that the annual loss of productivity due to their condition corresponded to 14, 21, and 28 days, respectively. In addition, we assumed that only caregivers of individuals with moderate (R2, M1) and severe (R3) diabetes-related visual impairment experienced annual productivity loss equivalent to 10 and 28 days, respectively. We used human capital method to measure the productivity lost. We multiplied the average wage per Saudi patient/caregiver by the total Productivity Lost (Absenteeism) annual Days for Mild/moderate/severe Vision Impaired Individual and the target population. The average wage per day of a Saudi patient was estimated using the Saudi gross domestic product (GDP) published by the World Bank for 2022 [23]. 

The Saudi government pensions given to individuals with severe (R4 and M2) diabetes-related visual impairment and blindness were obtained from published data [24]. Similarly, the average salary of homecare nurses in KSA was obtained from published data [25]. 

### Treatments

Patients with diabetic maculopathy (both M1 and M2) was treated by receiving 0.5 mg given as a single intravitreal injection of Anti-VEGF every 4 weeks and Triamcinolone Acetonide 4 mg once every 6 months. M1 patients perform optical coherence tomography (OCT) every 3 months, and every month they should have ophthalmologist visit, fundoscopic examination, visual field test + dilating eye drops, visual acuity, and fluorescein angiography - once at base time diagnosis, while M2 patients perform all of the above tests in addition to laser photocoagulation.

Patients with diabetic retinopathy (both R1, R2, R3, R4) received 0.3 mg of Anti-VEGF injected into the vitreous of the eye of every 4 weeks and Triamcinolone Acetonide 4 mg once every 6 months. R1 and R2 patients should perform Vitrectomy, and conduct post-operative tests every 3 months (OCT, ophthalmologist visit, fundoscopic examination, visual field test + dilating eye drops, visual acuity, and fluorescein angiography). R3 and R4 patients conduct all the above tests in addition to laser photocoagulation. M1, R1, R2, and R3 patients need Visual aids, while M2 and R4 patients need psychological support and home nurse visits (Supplementary data).

### Sensitivity analysis

A deterministic sensitivity analysis (DSA) within Microsoft Excel was performed to address uncertainties and assumptions in the study inputs and thereby ensure the robustness of the cost of illness model while identifying inputs with significant impact on the results. Inputs varied by ± 20%, and the DSA results were plotted in a tornado diagram, which shows the factors with the greatest impact on the total cost. The analysis highlighted the most sensitive input values that influenced the model’s results.

## Results

### Base case results

**Fig. 2:**
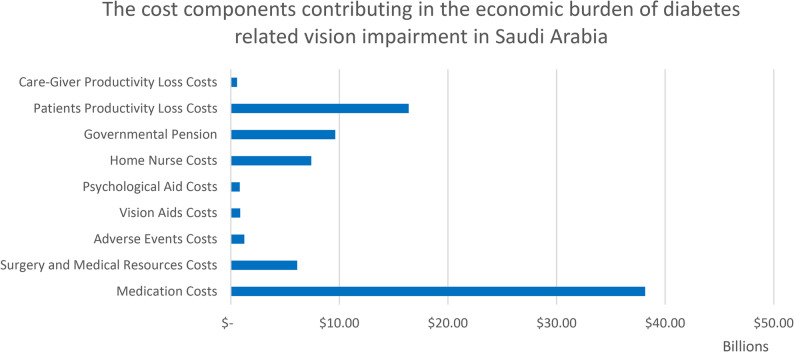
Base-Case resulted costs components

**Fig. 3:**
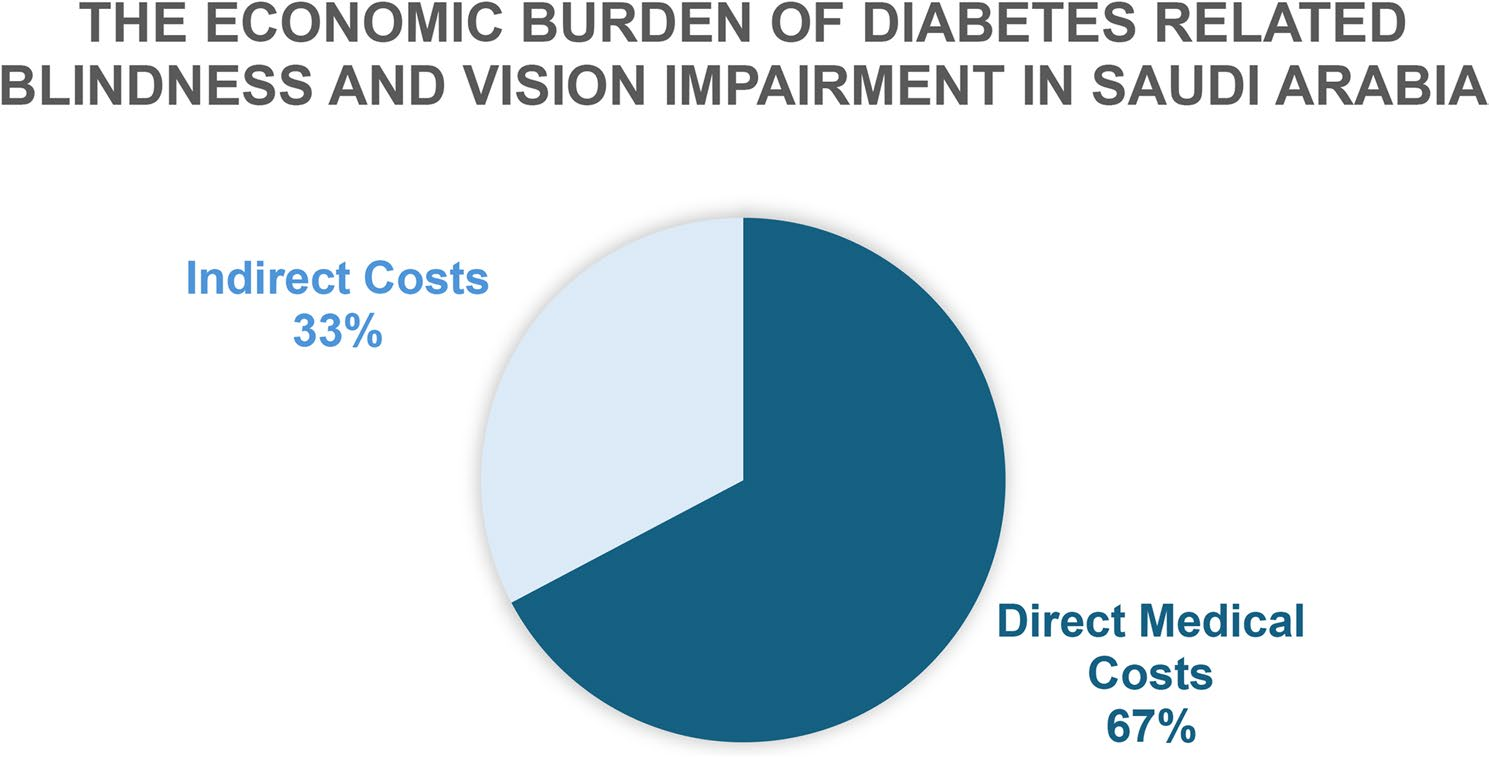
Base-Case resulted Cumulative Costs

Our economic cost of illness model showed that the total economic burden of visual impairment and blindness in KSA in one year (model’s time horizon) was SAR 150,324,912,186 ($ 81.2 billion), a huge burden on the economy representing 1.5% of the total GDP of KSA for 2024, impacting the whole country including patient, society, family and caregivers. This result highlights the huge economic burden faced by the healthcare system and government, which requires attention and urgent intervention. The main cost components that contribute to this huge economic burden on the population are the medication costs and the productivity loss costs (Fig. 3). The total cost comprised direct and indirect medical costs amounting to SAR 101,126,846,285 ($ 54.6 billion; 67%) and SAR 49,198,065,900 ($ 26.5 billion; 33%), respectively (Fig. 2). The total direct and indirect costs for each DR and DM grade and the total costs per each cost component are presented in Table 4.

**Table 4:** Base-Case Costs Break Down Results

Total Direct Medical Costs							Total Indirect costs			
Medication Costs	Surgery and Medical Resources Costs	Adverse Events Costs	Psychological Aid Costs		Vision Aids Costs	Home Nurse Costs	Governmental Pension	Patients Loss Productivity Costs		Care-Giver Costs
SAR 70,610,172,590	SAR 11,319,029,616	SAR 2,323,124,452	SAR 1,535,131,658		SAR 1,619,612,282	SAR 13,719,775,687	SAR 17,798,627,918	SAR 30,314,412,406		SAR 1,085,025,576
$ 38,167,660,859	$ 6,118,394,387.24	$ 1,255,742,947.19	$ 829,800,896.19		$ 875,466,098.18	$ 7,416,094,965.99	$ 9,620,879,955	$ 16,386,168,868		$ 586,500,311
DR/DM Grade	Direct Medical Costs per patient (SAR)			Direct Medical Costs per patient (I$)			Indirect costs per patient (SAR)		Indirect costs per patient (I$)	
DR 1	SAR 300,046			$ 162,186.95			SAR 145,972		$ 78,903.72	
DR 2	SAR 1,326,203			$ 716,866.34			SAR 645,196		$ 348,754.45	
DR 3	SAR 1,700,260			$ 919,059.40			SAR 827,174		$ 447,121.08	
DR 4	SAR 1,894,575			$ 1,024,094.77			SAR 921,708		$ 498,220.64	
DM 1	SAR 98,685			$ 53,343.03			SAR 13,568		$ 7,333.81	
DM 2	SAR 139,225			$ 75,256.54			SAR 162,180		$ 87,664.86	

*DR* Diabetic Retinopathy, *DM* Diabetic Maculopathy

### Sensitivity analysis results

**Fig. 4:**
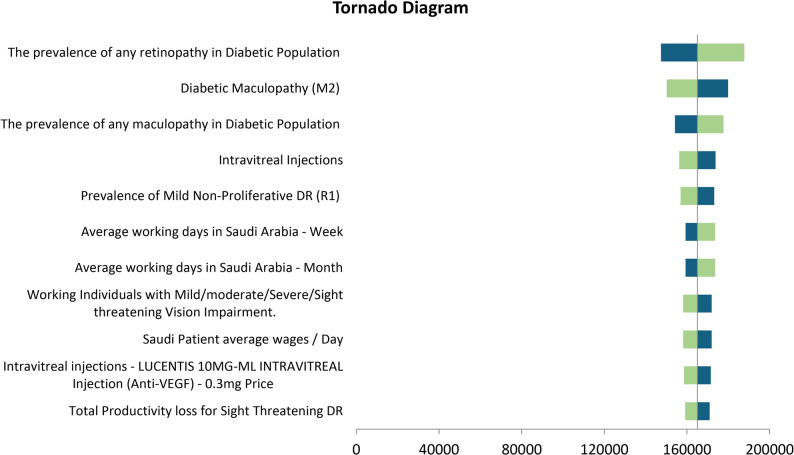
The results of one-way sensitivity analysis. Green colour refers to the output of the low value of the parameter, while the blue colour refers to the output of the high value of the parameter

One-way sensitivity analyses were conducted to test the robustness of the results. Figure 4 shows the tornado diagram, which demonstrates the results of the one-way sensitivity analyses. The most important parameter affecting the results was found to be the prevalence of retinopathy in the population with diabetes, which is a major factor contributing to the overall cost.

## Discussion

Rising diabetes rates and aging populations have increased the urgent need for DR data to help improve services and guide policymakers [19]. Visual impairment and blindness can have a wide range of economic impacts on individuals, households, and health systems. Our economic model estimated the one-year cost of diabetes-related visual impairment and blindness in KSA to be around SAR 150 billion (1.5% of total Saudi GDP; SAR 10 trillion). The highest expenditures were associated with medication costs and patients’ productivity loss. This highlights the importance of better resource allocation and investment in innovative treatments that could manage the diabetes-related visual impairment and reduce the huge burden paid by the country and imposed on the patient, caregiver, family, economy and the whole society.

A wide range of promotion, prevention, treatment, and rehabilitation strategies are available to address eye conditions and avoid visual impairment. They include measures for preventing vision loss caused by infections, trauma, unsafe traditional medicines, perinatal diseases, nutrition-related issues, and improper use of topical treatments. Early detection and timely treatment are crucial in conditions such as DR to avoid irreversible vision loss. Cost-effective treatments include spectacle correction for refractive errors and cataract surgery. However, globally, only 36% of individuals with distance vision impairment due to refractive errors have access to appropriate spectacles, and only 17% of those with visual impairment or blindness due to cataracts undergo quality surgery [26] because of limited access to these treatments.

Our results are in parallel with the published literature, which indicates that the economic impact of visual impairment and blindness includes significant productivity losses. Globally, visual impairment is estimated to cause an annual productivity loss of $411 billion. This loss is attributed to a reduction in employment of approximately 30.2% among people with moderate to severe visual impairment or blindness​. [27] In the United States, according to centers for disease control and prevention (CDC), major visual disorders such as age-related macular degeneration, cataracts, DR, and glaucoma result in a combined economic burden of $35.4 billion, which includes $8 billion in productivity losses [28]​ .

An American study analyzed secondary data sources that aimed to assess the economic burden of vision loss across the country in 2017 and included participants who reported blindness or serious difficulty seeing with glasses reported a total economic burden of $134.2 billion, with $98.7 billion in direct costs and $35.5 billion in indirect costs [29]. In Germany, the annual costs of blindness and visual impairment from a societal perspective amounted to € 49.6 billion [30].

An Ethiopian cross-sectional random study that included 425 individuals with an average age of 48 years and aimed to assess productivity loss among adults with visual impairment reported a total productivity loss of $775,325, which was primarily attributed to reduced workforce participation amounting to $746,337 [31]. These results highlight the extent to which vision loss can increase costs for both individuals and society in a low-income country. In Ecuador (middle income country), treating blindness caused by diabetic retinopathy and diabetic macular edema represents a yearly expense of $ 259.7 million − 92.4% can be attributed to productivity costs [32].

A study measuring the burden of vision loss in the MENA region, which utilized publicly available data from the Global Burden of Disease Study 2019 covering 21 countries in the MENA region, found that despite a decrease in the burden of vision loss over the last three decades in the region, it remains high, emphasizing the need for preventive measures, especially for older adults and low socioeconomic groups [33]. It was reported that the annual cost of lost productivity from moderate to severe vision impairment and blindness in the MENA region was estimated at $33.56 billion (0.35% of gross domestic product) [27].

One of the key recommendations that can help prevent diabetes-related vision impairment are prioritizing promotion and prevention that can help identify early warning signs of complications that could affect the vision. The second, investment in innovative treatments that could allocate resources and manage the diabetes-related visual impairment that could be new hope and support for patients with diabetes related eye conditions. These two recommendations align with the Saudi vision 2030 that introduces AI-Powered Early Detection of Diabetic Retinopathy to prevent diabetes-related eye diseases and enable early detection of diabetic retinopathy [34] .

Our study has several strengths, the first of which is the outset cost of illness analysis to evaluate visual impairment and blindness costs in KSA, focusing on differentiating between diabetic retinopathy and diabetic maculopathy. Second, to decrease uncertainty and strengthen the credibility of our results, we conducted a well-constructed sensitivity analysis for guaranteeing the robustness of the model. In addition, we validated all the inputs and assumptions used in our analysis through consultation with our Saudi ophthalmology expert panel.

Nevertheless, the study also has limitations. Owing to the limited availability of data, we relied on less evident data to capture the prevalence data and we relied as well on our local experts’ insights to determine the absenteeism days for individuals with mild and moderate visual impairment and their caregivers. In addition, we did not include other government aids given to those with severe visual impairment and blindness in KSA, such as transportation and rehabilitation aids, owing to the lack of data. Future local studies should address these limitations.

## Conclusion

The significant economic repercussions of diabetes-related visual impairment and blindness across individuals, and the Saudi healthcare system impose a substantial financial burden. The major component is medication costs, along with patients’ productivity losses. These findings highlight the urgent need for investment in innovative therapies and screening programs aimed at mitigating these costs, enhancing accessibility to essential treatments, improving the quality of life of affected individuals and families, and achieving sustainable healthcare and socioeconomic development goals in the Kingdom. Future research is in need to capture the real-world local data for diabetes-related complications across the country.

## Supplementary Information

Supplementary Material 1

Supplementary Material 2

## Data Availability

No datasets were generated or analysed during the current study.
