# Supplementary material for: The economic burden of diabetes-related visual impairment and blindness in Saudi Arabia
Source: Health Econ Rev. 2026 Jan 24;16:10. doi: 10.1186/s13561-026-00721-3 (PMC12837020; doi:10.1186/s13561-026-00721-3)
Supplement: Supplementary file 1 — Supplementary Material 1 [file 13561_2026_721_MOESM1_ESM.docx]

**Supplementary**

|  | | **Treatments and Managements Protocols within the Model** | | | | | |
| --- | --- | --- | --- | --- | --- | --- | --- |
| **#** | **Disease** | | **Treatment Protocol** | **Costs** | **Total Costs** |  |  |
| 1 | **Diabetic Maculopathy Assumed to occur in two eyes** | | **Intravitreal injections of Anti-VEGF:** Typically 0.3 mg injected into the vitreous of the eye every 4 weeks | SAR 35,308.80 |  |  |  |
|  |  |  | **Intravitreal injections of corticosteroids:** Triamcinolone Acetonide 4mg once every 6 months | SAR 1,180.20 |  |  |  |
|  |  |  | Intravitreal Injections | SAR 48,000.00 | **SAR 84,489.00** |  |  |
| 2 | **Diabetic Retinopathy Assumed to occur in the two eyes** | | **Intravitreal injections of Anti-VEGF:** Typically 0.3 mg injected into the vitreous of the eye every 4 weeks | SAR 35,308.80 |  |  |  |
|  |  |  | **Intravitreal injections of corticosteroids:** Triamcinolone Acetonide 4mg once every 6 months | SAR 1,180.20 |  |  |  |
|  |  |  | Intravitreal Injections | SAR 48,000.00 | **SAR 84,489.00** |  |  |
|  |  | |  |  |  |  |  |
|  |  | |  |  |  |  |  |
|  | | **Annual Resources Utilized and Follow-Up** | | | | | |
| **#** | **Disease** | | **Description** | **Costs** | **Total Costs** |  |  |
| 1 | **Diabetic Maculopathy Assumed to be in the two eyes** | | Laser Photocoagulation | SAR 2,400.00 |  |  |  |
|  |  |  | Optical coherence tomography (OCT) every 3 months | SAR 1,520.00 |  |  |  |
|  |  |  | Ophthalmologist Visit | SAR 4,140.00 |  |  |  |
|  |  |  | Fundoscopic examination | SAR 720.00 |  |  |  |
|  |  |  | Visual Field Test + Dilating Eye Drops | SAR 2,175.06 |  |  |  |
|  |  |  | Visual Acuity | SAR 2,160.00 | **Diabetic Maculopathy (M1)** | **Diabetic Maculopathy (M2)** |  |
|  |  |  | Fluorescein angiography - once at base time diagnosis | SAR 480.00 | **SAR 11,195.06** |  |  |
| 2 | **Diabetic Retinopathy Assumed to occur in the two eyes** | | Laser Photocoagulation | SAR 2,400.00 |  |  |  |
|  |  |  | Vitrectomy | SAR 6,800.00 | **Mild and Moderate DR (R1 and R2)** | **Severe NPDR and PDR (R3 and R4)** |  |
|  |  |  | **Post-operative Tests (done every 3 months):** Optical coherence tomography (OCT)  Ophthalmologist Visit Fundoscopic examination Visual Field Test + Dilating Eye Drops Visual Acuity Fluorescein angiography | SAR 6,505.02 | **SAR 13,305.02** | **SAR 15,705.02** |  |
|  |  | |  |  |  |  |  |
|  |  | |  |  |  |  |  |
|  | | **Adverse Events Management** | | | | | |
| **#** | **Disease** | | **Description** | **Costs** | **Total Costs** |  |  |
| 1 | **Endophthalmitis** | | Pars Plana Vitrectomy (PPV) | SAR 3,200.00 |  |  |  |
|  |  |  | Intravitreal Injection of Vancomycin (1mg/0.1 ml)+Ceftazidime (2.25mg/0.1ml) | SAR 2,096.70 |  |  |  |
|  |  |  | Optical coherence tomography (OCT) | SAR 380.00 |  |  |  |
|  |  |  | Aqueous and Vitreous Sampling | SAR 1,600.00 |  |  |  |
|  |  |  | Systemic Antibiotics (Vancomyscin 20mg/kg (pt average weight 70kg) every 8 hrs + Ceftazidime 2 gevery 8hrs for 7 days) | SAR 4,566 |  |  |  |
|  |  |  | Topical Antibiotics + Corticosteroids - Eye Drops for 7 days | SAR 33.00 |  |  |  |
|  |  |  | 2 Ward Hospitalization | SAR 1,200.00 | **SAR 13,076.15** |  |  |
| 2 | **Retinal Detachment** | | Pars Plana Vitrectomy (PPV) | SAR 3,200.00 | **SAR 3,200.00** |  |  |
| 3 | **Cataract** **Assumed to occur in the two eyes** | | Phacoemulsification surgery | SAR 8,800.00 |  |  |  |
|  |  |  | Intra-occular Lens | SAR 5,600.00 |  |  |  |
|  |  |  | **Pre-operative Tests:** Visual Accuity Refraction Test for selection of IOL Biometry (A-Scan) Ocular Surface Evaluation Pupil Size and Dynamics Corneal Topography Intra-occular Pressure Measurement | SAR 1,668.00 |  |  |  |
|  |  |  | **Post-operative Tests (done twice):** Ophthalmologist Visit Visual Accuity IOL Position and Stability Evaluation Ocular Surface Assessment Intra-occular Pressure Measurement | SAR 4,418.00 | **SAR 20,486.00** |  |  |
|  |  | |  |  |  |  |  |
|  |  | |  |  |  |  |  |
